# Distinct B Cell Subsets Changes as Potential Biomarkers of Response to Biologic Therapy in Crohn’s Disease

**DOI:** 10.3390/ijms26199539

**Published:** 2025-09-29

**Authors:** Anna Helmin-Basa, Maria Kopoń, Jarosław Koza, Edyta Strzyżewska, Aleksandra Skalska-Bugała, Fabian Leśniewski, Małgorzata Wiese-Szadkowska, Sara Balcerowska, Jacek Michałkiewicz, Maria Kłopocka

**Affiliations:** 1 Department of Immunology, Faculty of Farmacy, Collegium Medicum in Bydgoszcz, Nicolaus Copernicus University in Torun, 85-094 Bydgoszcz, Poland; edytaroszak96@gmail.com (E.S.); aleksandraaskalska@gmail.com (A.S.-B.); fabian.lesniewski@gmail.com (F.L.); malgorzatawiese@gmail.com (M.W.-S.); sara.balcerowska@onet.pl (S.B.); 2 Gastroenterology Clinic, Jan Biziel University Hospital No. 2, 85-168 Bydgoszcz, Poland; mariakopon@wp.pl (M.K.); jaroslaw.koza@cm.umk.pl (J.K.); maria.klopocka@cm.umk.pl (M.K.); 3 Department of Gastroenterology and Nutrition Disorders, Faculty of Health Science, Collegium Medicum in Bydgoszcz, Nicolaus Copernicus University in Torun, 85-168 Bydgoszcz, Poland; 4 Department of Microbiology and Clinical Immunology, The Children’s Memorial Health Institute, 04-730 Warsaw, Poland; jjmichalkiewicz@wp.pl

**Keywords:** B cell subsets, regulatory T cells, tumour necrosis factor-α inhibitors, α4β7 integrin inhibitors, Crohn’s disease

## Abstract

Biological therapies for Crohn’s disease (CD), including infliximab, adalimumab, and vedolizumab, show variable efficacy. While some predictive biomarkers exist, data on regulatory immune cells are limited. This study examined whether baseline levels of circulating T and B cell subsets can predict response to these treatments. We recruited 43 adults with conventional treatment-resistant active CD (CDAI > 330) and 16 healthy controls. Blood samples were analysed by flow cytometry at baseline (week 0) and after induction therapy (week 12 or 14, depending on the received drug) to measure T and B cell subsets and correlate them with disease activity. CD patients at baseline showed a significantly reduced frequency of memory B cells, CD5^+^CD1d^+^ B cells, plasmablasts, and transitional B cells. Additionally, significant negative correlations were identified between transitional B cells and calprotectin/platelets, and between CD5^+^CD1d^+^ B cells and calprotectin. All CD patients responded clinically to biologic therapy. In those treated with infliximab or adalimumab, mature naïve B cells decreased, with a trend toward increased CD24^hi^CD27^+^ B cells. Adalimumab responders showed a trend toward higher CD161 expression on Tregs, while vedolizumab-treated patients had a slight increase in plasmablasts. Biologic therapies in CD revealed treatment-specific immune correlations: infliximab/adalimumab responses involved B and T cell changes linked to inflammation, while VDZ response correlated with CD4^+^ and CD5^+^CD1d^+^ B cells. Our study suggests that infliximab/adalimumab induction therapy in CD expands circulating CD24^hi^CD27^+^ B cells and reduces mature naïve B cells, while vedolizumab increases plasmablasts. These B-cell changes may reflect distinct mechanisms and serve as potential response biomarkers.

## 1. Introduction

The inflammatory bowel diseases (IBD), such as Crohn’s disease (CD), are multifactorial chronic inflammatory diseases involving the gastrointestinal tract. The aetiology of CD is still not well-known. The incidence and prevalence of CD vary across geographic regions, with the highest epidemiological burden observed in Europe, Oceania, and North America. The prevalence in Europe ranges from 1.5 to 331 per 100,000 people and is the highest in Germany. The mortality rate of patients diagnosed with CD has been shown to exceed that of the general population (standardised mortality ratio 1.4) [1,2]. The development of CD is determined by a variety of factors. Genetic and environmental factors are known to be decisive. The genetic mechanism in IBD involves inheritance of certain irregularities in immunological response that, in the presence of environmental factors, may lead to the development of CD. Certain variants in pattern recognition and barrier genes (e.g., NOD2 [3], ATG16L1 [4]), cytokine pathway genes (IL-23R [5,6,7]), STAT3, JAKs [8,9]) as well as antigen-presentation and innate immune genes, alter microbe recognition and cytokine production by antigen-presenting cells and epithelial cells. These changes support proinflammatory T helper (Th) 17 expansion and/or impair anti-inflammatory regulatory CD4^+^ T cells (Tregs). Environmental factors such as dysbiosis [10,11], diet, smoking, antibiotics, and enteric infections also modify the local cytokine milieu, metabolites, and antigen exposure, shifting the balance toward Th17. The imbalance between the proinflammatory Th17 and anti-inflammatory Tregs differentiation plays a significant role in the pathogenesis of abnormal intestinal immunity in CD [12] and promotes the action of α4β7 and αEβ7 integrins, tumour necrosis factor α (TNF-α), IL-1β, IL-6 and IL-23 what induce the development of mucosal inflammation [13]. Integrins are cell adhesion molecules on leukocytes that bind to addressins on gut endothelial cells. A high level of α4β7 integrin on effector/memory T and B cells increases their trafficking to the gut [14,15]. Upregulation of αEβ7 integrin on mucosal proinflammatory CD4^+^ T cells leads to their retention in mucosal tissue, enhancing immune activation [16,17,18]. Overproduction of proinflammatory TNF-α leads to increased intestinal permeability [19,20,21] and ulceration of the mucosa [22], as well as upregulation of other proinflammatory cytokines such as IL-1β, IL-6 [23,24], and IL-23 [25], which favour the expansion of Th17 cells [5,26]. Targeted biologic therapies in CD have been developed to interrupt these pathogenic cascades by selectively neutralising critical cytokines or inhibiting integrin-mediated leukocyte trafficking to the gut mucosa. The effectiveness of biologics is generally good [27,28,29,30,31,32,33,34,35,36,37,38], but variable depending on factors such as biologic class, disease location, severity and duration, presence of anti-drug antibodies, smoking, previous surgery, patient’s immunological profiles, treatment history, genetic factors, and treatment goals [39,40,41,42,43,44,45,46,47,48,49,50,51,52,53,54,55,56,57,58].

Anti-TNF-α antibodies, including infliximab (IFX) or adalimumab (ADA), and the gut-selective anti-4β7 integrin, such as vedolizumab (VDZ), are the biologics used in clinical practice for CD treatment. IFX and ADA are the most well-documented biologics to strongly induce remission, steroid-free remission, and maintenance of remission [59,60,61,62]. However, one-third of IFX or ADA recipients will show primary nonresponse, while another one-third will lose the response during the treatment [63]. The efficacies of these agents are difficult to predict. A few biomarkers were identified in relation to anti-TNF-α agents and VDZ responses. Many studies have reported predictors of biological efficacy among immune factors. Genes associated with recognition of bacterial components and cytokine signalling could predict response to IFX or ADA [64]. The expression of integrin α4β1 [65] and the peripheral blood level of IL-6 [66] were identified as potential biomarkers of VDZ response. However, there is a lack of new decisive immunological predictors among immune cells that could help in choosing the best therapeutic option. Interestingly, B and T cells with regulatory phenotypes may be considered as potential candidates. There are early and somewhat inconsistent findings that certain mucosal and peripheral blood Treg phenotypes may help predict which CD patients will respond to anti-TNF-α therapies [67,68] or to VDZ [69]. However, there is currently no evidence that Bregs can serve as predictors of response to biologic therapy.

Regulatory B cells (Bregs) have special immune regulatory functions to modulate inflammation and autoimmunity. Breg population uses different immunosuppressive mechanisms: anti-inflammatory cytokines such as IL-10 [70,71], IL-35 [72,73] and transforming growth factor β (TGF-β) [74,75], as well as cell surface proteins like CD1d [76,77,78] and PDL1 [79,80], ectonucleotides (CD39/CD73) [81,82] or non-inflammatory isotypes (IgG4) [83,84] or IgA [85,86,87,88,89]. Different Breg subsets used overlapping but distinct immunosuppressive mechanisms [90,91]. They influence immune regulation by activating Tregs, regulating Th1/Th2 cytokine balance and dendritic cell functions, downregulating the proinflammatory network, and suppressing T cell-mediated autoimmunity [92,93,94], including animal models of spontaneous colitis [95,96,97]. In autoimmune disorders, the loss of Breg frequency or functional impairment has been observed, but the dominant defective mechanisms depend on the disease [98,99]. A lack or dysfunction of Bregs as well as Treg cells leads to a worsening course of autoimmune diseases, including CD [100,101]. Restoring functional Bregs in CD could directly dampen Th1/Th17 inflammation and support Treg expansion, promoting mucosal healing.

There is still a lack of a set of phenotypic markers or transcriptional factors that can be used to identify Bregs [86,92,102,103,104,105]. However, phenotypically distinct populations of human B cells have been identified that exhibit regulatory activity under specific stimulatory conditions. IL-10^+^ Bregs can already be found among CD24^hi^CD38^hi^ transitional B cells [106,107], CD24^hi^CD27^+^ memory B cells [108], CD5^+^ B cells [109,110,111], and CD5^+^CD1d^+^ B cells [112,113,114] as well as antibody-secreting cells such as plasmablasts and terminally differentiated plasma cells [104,115,116]. To the best of our knowledge, human Bregs are a combination of phenotypic markers, functional indicators (IL-10, TGF-β, IL-35), and transcriptional variables (transcripts of IL-10, BLIMP1, IRF4, AHR), and immune checkpoints) [102,117].

Recently, several different subsets of Treg have also been identified: FoxP3^+^CD25^+^CD127^−^ natural Tregs [118] arising in early ontogeny in the thymus, induced Tregs arising by induction in the periphery, memory Tregs defined by lack of CD45RA expression, naive Tregs with CD45RA expression [119] and proinflammatory CD161 lectin-like receptor^+^ Tregs [120].

Some immunological features of Tregs and Bregs, such as absolute or relative frequency, phenotype, suppression function, transcriptional and metabolic signatures, stability markers, and tissue homing, may be linked to treatment outcomes in CD patients receiving biological therapies [69,101,121,122,123,124].

Previous studies have shown that the proportion of Bregs is slightly increased [100,101], whereas the Treg pool is reduced in patients with active CD [76,100,101,125,126,127]. Additionally, in comparison to non-responders, responders tend to have higher blood or mucosal Treg abundance, greater Treg suppressive function, and more stable Treg lineage features [67,69,128,129]. The proportion of Bregs also increased or returned to near normal levels in responders [101,121]. Therefore, the aim of the present study was to assess whether the baseline proportions of circulating B cell and T subpopulations with regulatory phenotypes could help to identify CD patients who are likely to respond to anti-TNF-α agents (IFX and ADA) or to VDZ.

## 2. Results

### 2.1. Disease Activity Markers in All Recruited Patients with CD upon Entry into Study

Disease activity markers—including CD activity index (CDAI), serum C-reactive protein (CRP), faecal calprotectin, and platelet count—were assessed in CD patients at study entry (Table 1). The median CDAI was 330 [316.0; 381.0], the median CRP was 4.80 mg/L [1.60; 17.0], the faecal calprotectin median was 613.0 μg/g [112.0; 2715.0], and the median platelet count was 311 × 10^9^/L [273.0; 384.0].

### 2.2. Reduced Pool of Total Lymphocytes and Increased CD161 Expression on Tregs in Peripheral Blood of Patients with Active CD

**Figure 1:**
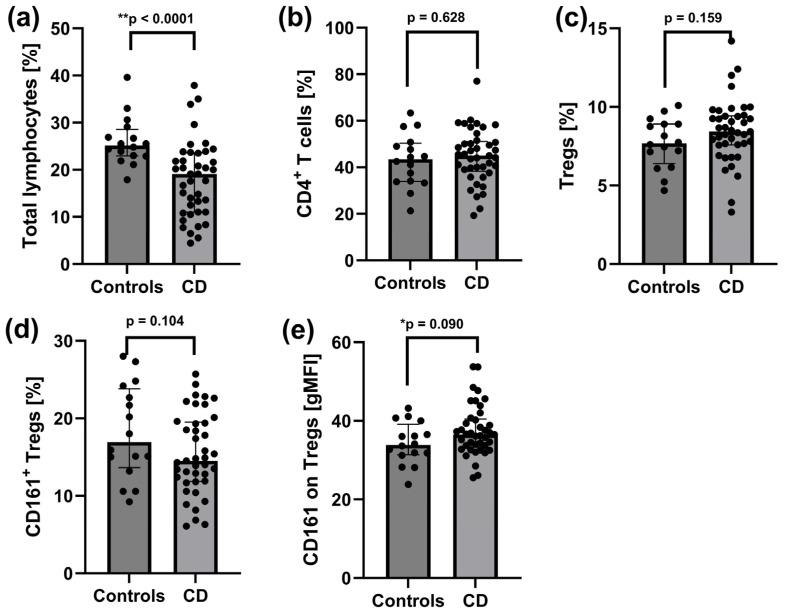
Distribution of total lymphocytes, CD4^+^ T cells, Tregs, and CD161^+^ Tregs in the peripheral blood of patients with active CD (*n* = 43) and controls (*n* = 16). (**a**) Frequency of total lymphocytes. (**b**) Frequency of CD4^+^ T cells. (**c**) Frequency of Tregs (CD4^+^CD25^hi^CD127^−^). (**d**) Frequency of CD161^+^ Tregs (CD4^+^CD25^hi^CD127^−^CD161^+^). (**e**) Surface expression of CD161 receptor on Tregs. Statistical analysis was performed using the Mann–Whitney U test: ** *p* < 0.05: statistically significant differences; * *p* < 0.1: tendency toward statistical significance. Individual group comparisons were conducted using linear contrasts. Each black dot on the graph represents an individual patient’s result.

The frequency of total lymphocytes in peripheral blood was significantly lower in patients with active CD compared to healthy controls (*p* < 0.0001) (Figure 1a). No significant differences were observed in the proportions of total CD4^+^ lymphocytes (Figure 1b), total Tregs (Figure 1c), or CD161^+^ Tregs (Figure 1d). However, the expression level of the CD161 surface receptor on peripheral Tregs showed a trend toward an increase in CD patients (*p* = 0.090) (Figure 1e).

### 2.3. Decreased Frequencies of Memory B Cells, CD5^+^CD1d^+^ B Cells, CD27^int^CD38^hi^ Plasmablasts, and CD24^hi^CD38^hi^ Transitional B Cells in Peripheral Blood of Patients with Active CD

**Figure 2:**
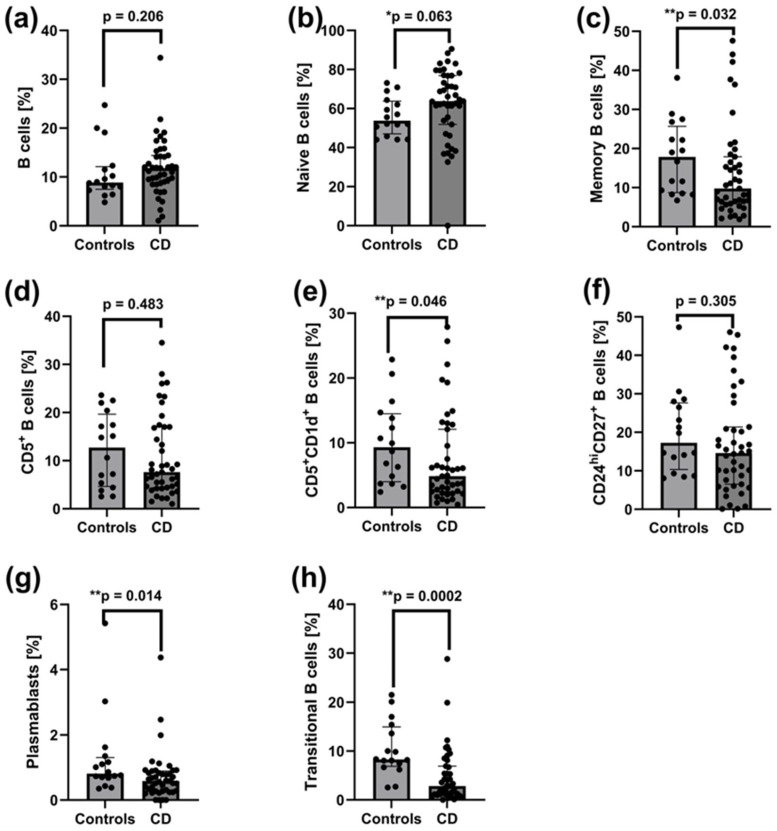
Distribution of total B cells and their subsets in the peripheral blood of patients with active CD (*n* = 43) and healthy controls (*n* = 16). (**a**) Frequency of total B cells (CD19^+^). (**b**) Frequency of mature naive B cells (CD24^int^CD38^int^CD19^+^). (**c**) Frequency of memory B cells (CD24^hi^CD38^−^CD19^+^). CD19^+^), (**d**) Frequency of CD5^+^ B cells (CD5^+^CD19^+^). (**e**) Frequency of CD5^+^CD1d^+^ B cells (CD5^+^CD1d^+^CD19^+^). (**f**) Frequency of CD24^hi^CD27^+^ B cells (CD24^hi^CD27^+^CD19^+^). (**g**) Frequency of plasmablasts (CD27^int^CD38^hi^CD19^+^). (**h**) Frequency of transitional B cells (CD24^hi^CD38^hi^CD19^+^). Statistical analysis was performed using the Mann–Whitney U test: ** *p* < 0.05: statistically significant differences; * *p* < 0.1: tendency toward statistical significance. Individual group comparisons were conducted using linear contrasts. Each black dot on the graph represents an individual patient’s result.

No significant differences were observed in the frequencies of total CD19^+^ B cells (Figure 2a), naïve B cells (Figure 2b), CD5^+^ B cells (Figure 2d), or CD24^hi^CD27^+^ regulatory B cells (Figure 2f) in peripheral blood between patients with active CD and healthy controls. However, patients with active CD showed significantly reduced frequencies of memory B cells (*p* = 0.032) (Figure 2c), CD5^+^CD1d^+^ B cells (*p* = 0.046) (Figure 2e), CD27^int^CD38^hi^ plasmablasts (*p* = 0.014) (Figure 2g), and CD24^hi^CD38^hi^ transitional B cells (*p* = 0.0002) (Figure 2h) compared to healthy controls.

### 2.4. Association of T and B Cell Subset Frequencies with the Disease Activity Markers (i.e., Crohn’s Disease Activity Index, C-Reactive Protein, Faecal Calprotectin, and Platelet Count) in Patients with Active CD

In active CD patients, several significant correlations were observed: (a) transitional B cells negatively correlated with faecal calprotectin and platelet count (*r* = −0.392 and *r* = −0.309, respectively, *p* < 0.05), (b) CD5^+^CD1d^+^ B cells inversely correlated with calprotectin (*r* = −0.356, *p* < 0.05), (c) Tregs negatively correlated with CDAI (*r* = −0.390, *p* < 0.05), and (ed) CD5^+^ B cells inversely correlated with faecal calprotectin and platelet counts (*r* = −0.393 and *r* = −0.328, respectively, *p* < 0.05). There was not any significant correlation between B cell subset frequencies and CDAI (total B cells: *r* = −0.08, naïve B cells: *r* = −0.07, memory B cells: *r* = 0.006, CD5^+^ B cells: *r* = −0.27, CD5^+^CD1d^+^ B cells: *r* = −0.287, CD24^hi^CD27^+^ B cells: *r* = 0.04, plasmablasts: *r* = 0.07, and transitional B cells: *r* = −0.181, *p* > 0.05).

### 2.5. Comparable Disease Activity Markers Between Pre-Treatment CD Patient Groups

CDAI, serum CRP, faecal calprotectin, and platelet count were assessed in CD patients before the first dose of biological treatment with adalimumab (ADA), infliximab (IFX), and vedolizumab (VDZ) (Table 2). There were no significant differences in any disease activity markers between pre-treatment CD patient groups (*p* > 0.05, U-Mann–Whitney test).

### 2.6. Reduced Total Lymphocyte Pool and Increased CD161 Expression on Tregs in Peripheral Blood of CD Patients Prior to IFX, ADA, and VDZ Treatment

**Figure 3:**
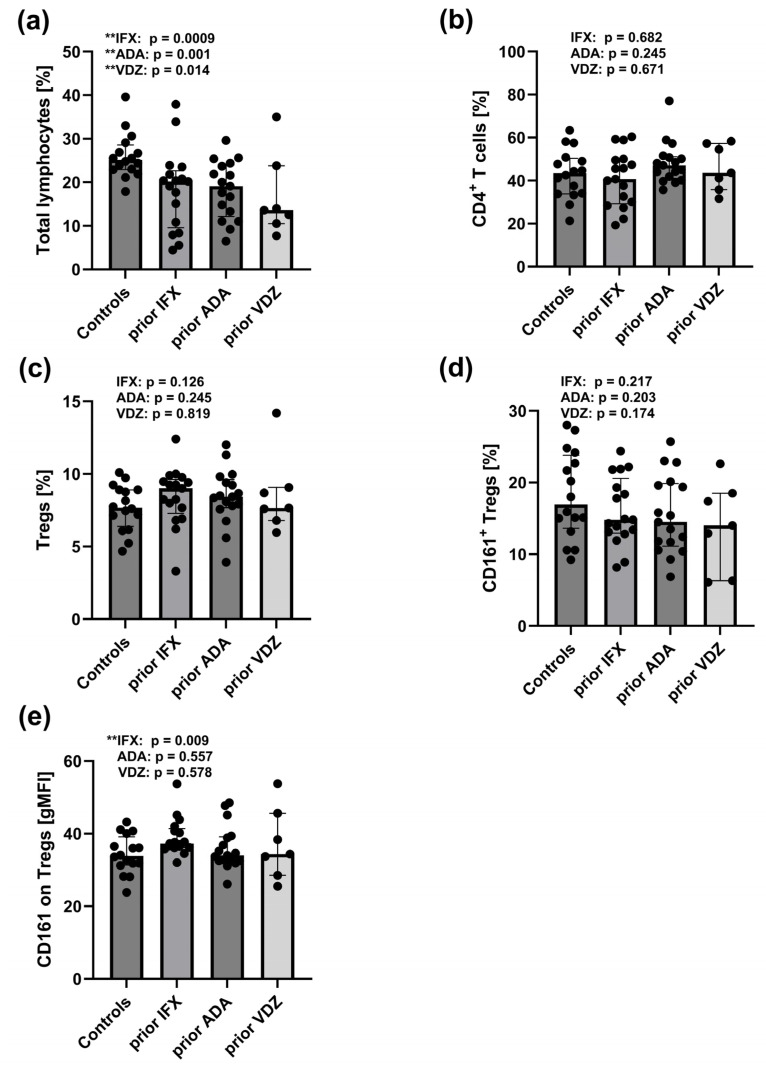
Distribution of total lymphocytes, CD4^+^ T cells, Tregs, and CD161^+^ Tregs in the peripheral blood of healthy controls (*n* = 16) and CD patients prior to treatment with IFX (*n* = 17), ADA (*n* = 17), and VDZ (*n* = 7); (**a**) Frequency of total lymphocytes. (**b**) Frequency of CD4^+^ T cells. (**c**) Frequency of Tregs (CD4^+^CD25^hi^CD127^−^). (**d**) Frequency of CD161^+^ Tregs (CD4^+^CD25^hi^CD127^−^CD161^+^). (**e**) Surface expression of CD161 receptor on Tregs. Statistical analysis was performed using the Mann–Whitney U test: ** *p* < 0.05: statistically significant differences: IFX: prior IFX versus controls, ADA: prior ADA versus controls, and VDZ: prior VDZ versus controls. Each black dot on the graph represents an individual patient’s result.

The total lymphocyte pool was significantly lower in CD patients prior to treatment with ADA (*p* = 0.001), IFX (*p* = 0.0009), and VDZ (*p* = 0.014) compared to healthy controls (Figure 3a). In contrast, the surface expression of the CD161 receptor on Tregs cells was increased, particularly in patients prior to IFX treatment (*p* = 0.014) (Figure 3e). However, no significant differences were observed in the frequencies of CD4^+^ T cells (Figure 3b), total Tregs (Figure 3c), or CD161^+^ Tregs (Figure 3d) between pre-treatment CD patient groups and controls.

### 2.7. Reduced Frequencies of CD27^int^CD38^hi^ and CD24^hi^CD38^hi^ B Cell Subsets in Peripheral Blood of CD Patients Prior to IFX, ADA, and VDZ Treatment

**Figure 4:**
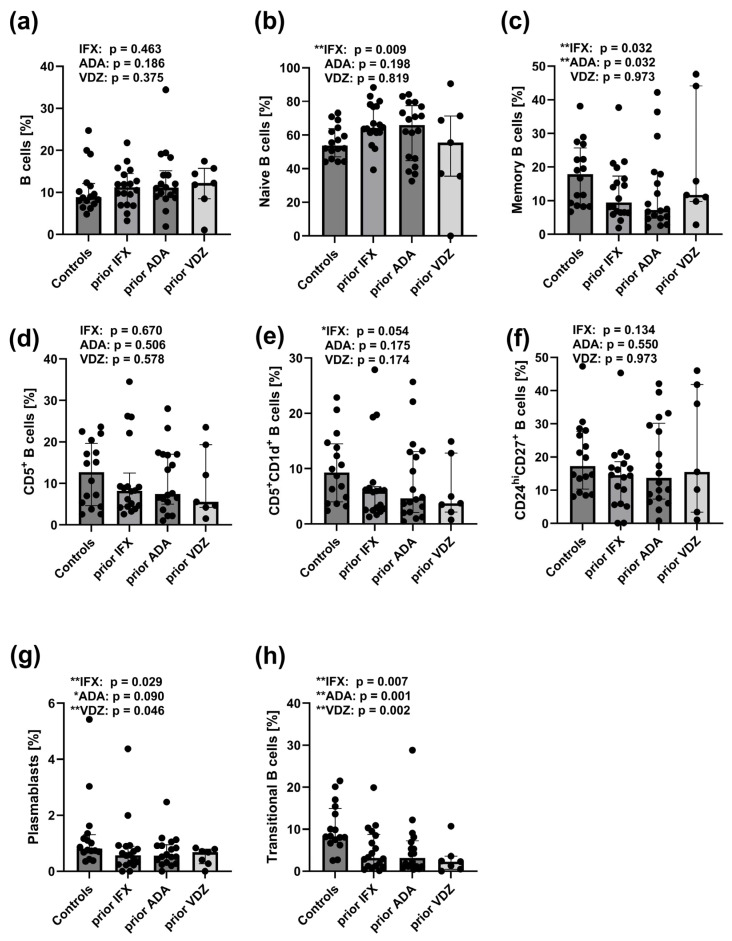
Distribution of total B cells and their subsets in the peripheral blood of healthy controls (*n* = 16) and CD patients prior to treatment with IFX (*n* = 18), ADA (*n* = 18), and VDZ (*n* = 7). (**a**) Frequency of total B cells (CD19^+^). (**b**) Frequency of mature naive B cells (CD24^int^CD38^int^CD19^+^). (**c**) Frequency of memory B cells (CD24^hi^CD38^−^CD19^+^), (**d**) Frequency of CD5^+^ B cells (CD5^+^CD19^+^). (**e**) Frequency of CD5^+^CD1d^+^ B cells (CD5^+^CD1d^+^CD19^+^). (**f**) Frequency of CD24^hi^CD27^+^ B cells (CD24^hi^CD27^+^CD19^+^). (**g**) Frequency of plasmablasts (CD27^int^CD38^hi^CD19^+^). (**h**) Frequency of transitional B cells (CD24^hi^CD38^hi^CD19^+^). Statistical analysis was performed using the Mann–Whitney U test: ** *p* < 0.05: statistically significant differences, * *p* < 0.01: tendency toward statistical significance: IFX: prior IFX versus controls, ADA: prior ADA versus control, and VDZ: prior VDZ versus controls. Each black dot on the graph represents an individual patient’s result.

The frequencies of memory B cells (Figure 4c), CD27^int^CD38^hi^CD19^+^ plasmablast (Figure 4g), and CD24^hi^CD38^hi^CD19^+^ transitional B cells (Figure 4h), were significantly reduced in CD patients prior to treatment with ADA (*p* = 0.032, *p* = 0.090, and *p* = 0.001, respectively) and IFX (*p* = 0.032, *p* = 0.029 and *p* = 0.007, respectively). In VDZ pre-treatment CD patients, only plasmablast (Figure 4g) and transitional B cell (Figure 4h) frequencies were decreased (*p* = 0.046 and *p* = 0.002, respectively). An increased frequency of mature naive B cells was observed exclusively in CD patients prior to IFX treatment (*p* = 0.009) (Figure 4b). No significant differences were found in the remaining B cell subsets between pre-treatment CD patient groups and healthy controls (Figure 4a,d–f).

### 2.8. Association of T and B Cell Subset Frequencies with the Disease Activity Markers (i.e., Crohn’s Disease Activity Index, C-Reactive Protein, Faecal Calprotectin and Platelet Count) in CD Patient Groups Before Biological Therapy

In CD patients prior to IFX treatment, CDAI negatively correlated with the frequencies of CD4^+^ T cells and Tregs (*r* = −0.497 and *r* = −0.517, respectively; *p* < 0.05, whereas a positive correlation was observed with the pool of CD27^int^CD38^hi^ B cells (*r* = 0.478, *p* < 0.05). CRP levels showed a negative correlation with the proportion of total lymphocytes (*r* = −0.528, *p* < 0.05). Furthermore, faceal calprotectin negatively correlated with the pool of CD5^+^ B cells and CD5^+^CD1d^+^ B cells (*r* = −0.536 and *r* = −0.643, respectively; *p* < 0.05).

In CD individuals prior to ADA therapy, CDAI negatively correlated with the frequencies of CD5^+^ and CD5^+^CD1d^+^ B cells (*r* = −0.536 and *r* = −0.592, respectively; *p* < 0.05).

In CD patients before VDZ treatment, negative correlations were observed between CDAI and CD4^+^ T cells (*r* = −0.942, *p* < 0.05), as well as between faecal calprotectin and CD24^hi^CD38^hi^ B cells (*r* = −0.857, *p* < 0.05). Additionally, a positive correlation was found between CDAI and memory B cells (*r* = 0.821, *p* < 0.05).

### 2.9. Decreased Disease Activity Markers Following Induction Therapy with ADA, IFX, and VDZ

**Figure 5:**
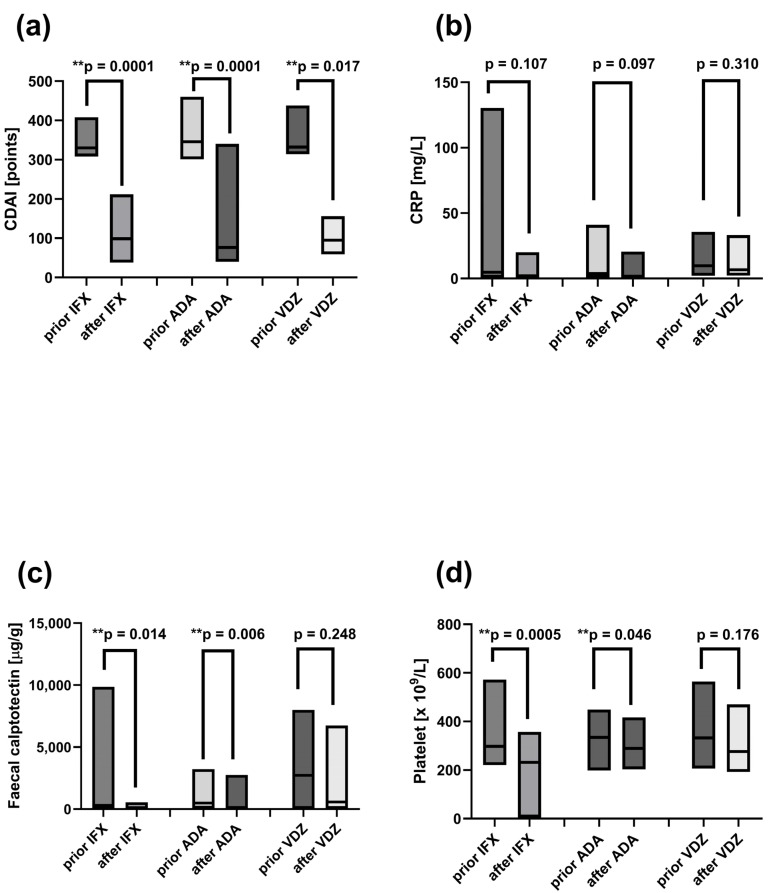
Disease activity markers in CD patients before and after induction therapy with infliximab (IFX), adalimumab (ADA), or vedolizumab (VDZ). The markers include (**a**) Crohn’s Disease Activity Index (CDAI), (**b**) C-reactive protein (CRP), (**c**) faecal calprotectin, and (**d**) platelet count. Statistical analysis was performed using the Wilcoxon matched-pairs test: ** *p* < 0.05: statistically significant differences. Individual group comparisons were conducted using linear contrasts.

Disease activity markers—including CDAI, CRP, faecal calprotectin and platelet count—were assessed in CD patients before the first dose of biological treatment and after induction therapy with ADA, IFX and VDZ (Figure 5). CDAI scores significantly decreased following induction therapy with ADA (at week 12; *p* = 0.0001), IFX (at week 14; *p* = 0.0001), and VDZ (at week 14; *p* = 0.017) (Figure 5a). Clinical remission—defined as a reduction of 70 points in CDAI from baseline value and at least a 25% decrease from baseline total score —was achieved in all patients after induction therapies. Significant reduction in faecal calprotectin (Figure 5c) and platelet count (Figure 5d) was observed after induction treatment with ADA (*p* = 0.006 and *p* = 0.046, respectively) and IFX (*p* = 0.014 and *p* = 0.0005, respectively). No significant changes in CRP levels were detected during the study period (Figure 5b).

### 2.10. Increased Frequency of Peripheral Lymphocytes and CD161 Receptor on Tregs Following Induction Therapy with ADA and IFX

The frequencies of peripheral total lymphocytes, CD4^+^ T cells, and their subsets were compared in CD before and after induction therapy with ADA, IFX, or VDZ.

**Figure 6:**
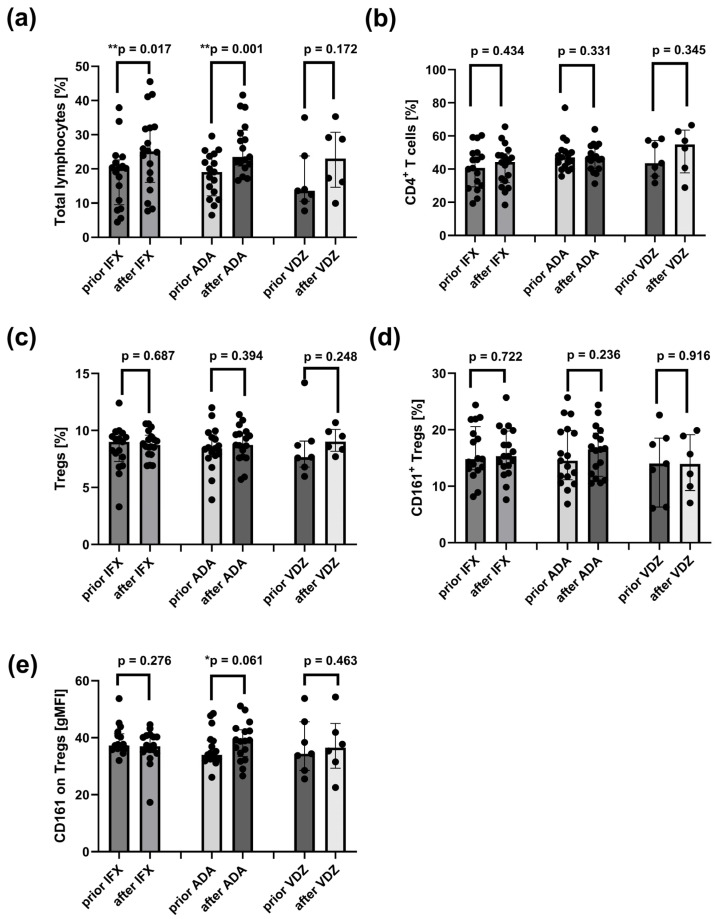
Distribution of total lymphocytes, CD4^+^ T cells, Tregs and CD161^+^ Tregs in the peripheral blood in CD patients prior and after treatment with IFX (*n* =17), ADA (*n* =17), and VDZ (*n* = 7). (**a**) Frequency of total lymphocytes. (**b**) Frequency of CD4^+^ T cells. (**c**) Frequency of Tregs (CD4^+^CD25^hi^CD127^−^). (**d**) Frequency of CD161^+^ Tregs (CD4^+^CD25^hi^CD127^−^CD161^+^). (**e**) Surface CD161 expression on Tregs. Statistical analysis was performed using the Wilcoxon matched-pairs test: ** *p* < 0.05: statistically significant differences, * *p* < 0.1: tendency to statistical significance. Individual group comparisons were conducted using linear contrasts. Each black dot on the graph represents an individual patient’s result.

A significant increase in total lymphocyte frequency was observed after treatment with ADA (*p* = 0.001) and IFX (*p* = 0.017) compared to pre-treatment values (Figure 6a). The surface expression of CD161 receptor on peripheral Tregs showed a trend toward an increase following ADA therapy (*p* = 0.061) (Figure 6e). However, no significant differences were observed in the frequencies of CD4^+^ T cells, total Tregs, or CD161^+^ Tregs before and after therapy with ADA, IFX, or VDZ (Figure 6b–d).

### 2.11. Reduced Frequencies of Mature Naïve B Cells and Increased Proportions of CD24^hi^CD27^+^ B Cells in CD Patients After Induction Therapy with ADA and IFX

The frequencies of peripheral CD19^+^ B cells and their subsets were compared in CD patients before and after induction therapy with ADA, IFX, or VDZ.

**Figure 7:**
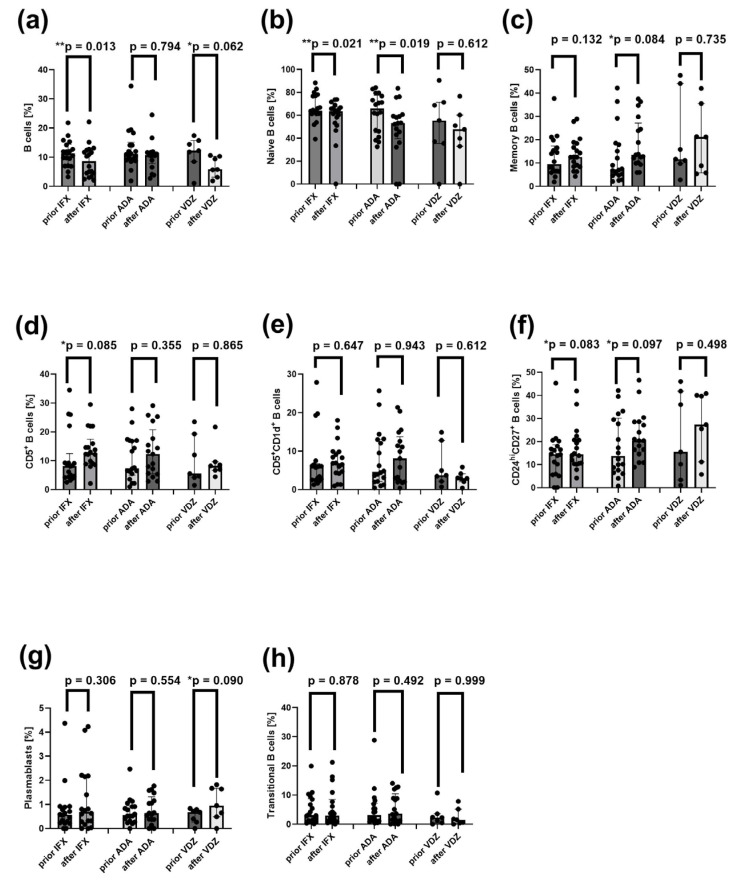
Distribution of total B cells and their subsets in the peripheral blood in CD patients before and after treatment with IFX (*n* =17), ADA (*n* =17), and VDZ (*n* = 7). (**a**) Frequency of total B cells (CD19^+^) (**b**) Frequency of mature naive B cells (CD24^int^CD38^int^CD19^+^). (**c**) Frequency of memory B cells (CD24^hi^CD38^−^CD19^+^), (**d**) Frequency of CD5^+^ B cells (CD5^+^CD19^+^). (**e**) Frequency of CD5^+^CD1d^+^ B cells (CD5^+^CD1d^+^CD19^+^). (**f**) Frequency of CD24^hi^CD27^+^ B cells (CD24^hi^CD27^+^CD19^+^). (**g**) Frequency of plasmablasts (CD27^int^CD38^hi^CD19^+^). (**h**) Frequency of transitional B cells (CD24^hi^CD38^hi^CD19^+^). Statistical analysis was performed using the Wilcoxon matched-pairs test: ** *p* < 0.05: statistically significant differences, * *p* < 0.1: tendency to statistical significance. Individual group comparisons were conducted using linear contrasts. Each black dot on the graph represents an individual patient’s result.

Total CD19^+^ B cells (Figure 7a) and mature naïve B cells (Figure 7b) were significantly reduced after ADA (*p* = 0.006 and *p* = 0.019) and IFX (*p* = 0.013 and *p* = 0.021) treatments. In contrast, the frequency of CD24^hi^CD27^+^ regulatory B cells tended to increase after ADA (*p* = 0.097) and IFX *(p* = 0.083), but not after VDZ (*p* = 0.498) (Figure 7f). Additionally, the frequency CD27^int^CD38^hi^ B cells (plasmablasts) was elevated only in CD patients after VDZ treatment (*p* = 0.090) (Figure 7g). No significant differences were observed in the remaining B cell subsets between patients before and after induction therapies (Figure 7c–e,h).

### 2.12. Association of T and B Cell Subset Frequencies with the Disease Activity Markers (i.e.,Crohn’s Disease Activity Index, C-Reactive Protein, Faecal Calprotectin and Platelet Count) in CD Patient After Biological Therapy

In IFX-treated CD patients, the following correlations were observed: (1) a negative correlation between CDAI and the frequency of T CD4^+^ cells (*r* = −0.504, *p* < 0.05), as well as between platelet count and CD5^+^CD1d^+^ B cells (*r* = −0.525, *p* < 0.05); (2) a positive correlation between CDAI and the pool of CD24^hi^CD38^hi^ B cells (*r* = 0.620, *p* < 0.05).

In CD patients after ADA treatment, CDAI negatively correlated with the pool of proinflammatory Tregs (*r* = −0.575, *p* < 0.05), but positively with CD27^int^CD38^hi^ B cells (*r* = 0.515, *p* < 0.05). Additionally, CRP and faecal calprotectin negatively correlated with the frequency of total B cells (*r* = −0.529 and *r* = −0.602, respectively; *p* < 0.05).

In VDZ-treated CD patients, feacel calprotectin negatively correlated with the frequency of CD4^+^ T cells (*r* = −0.942, *p* < 0.05), but positively with the pool of CD5^+^CD1d^+^ B cells (*r* = −0.785, *p* < 0.05).

## 3. Discussion

All 43 patients with Crohn’s disease (CD) included in this study demonstrated a significant reduction in Crohn’s Disease Activity Index (CDAI) scores following induction therapy with adalimumab (ADA) at week 12, and with infliximab (IFX) or vedolizumab (VDZ) at week 14. A clinical response was observed in all enrolled patients. In those treated with ADA or IFX, improvements were also seen in biochemical markers of inflammation, including faecal calprotectin and platelet count, although C-reactive protein (CRP) levels remained unchanged.

### 3.1. Altered B-Cell Subsets and Increased CD161 Expression on Tregs at Baseline

To characterise the immunological profile of these patients at baseline, we compared peripheral blood lymphocyte distributions—both total and subset populations of T and B cells (naïve, memory, and those with regulatory phenotypes)—between patients with active CD who had failed conventional therapy and age- and sex-matched healthy controls. Compared to controls, CD patients exhibited a reduced frequency of circulating memory B cells, CD5^+^CD1d^+^ B cells, plasmablasts, and transitional B cells. Conversely, mature naïve B cells and surface expression of the CD161 receptor on regulatory T cells (Tregs) were slightly increased. All patients included in this analysis presented with a severe disease flare according to CDAI and had previously received standard treatments, including 5-aminosalicylic acid (5-ASA), corticosteroids, and immunosuppressants.

Our observation of a reduced pool of CD24^hi^CD38^−^ memory B cells is consistent with previous studies reporting decreased memory B cells [130] and their subsets, including IgM memory B cells [121,131,132], CD27^+^IgD^−^ switched memory B cells, and CD27^−^IgD^−^ B cells expressing all immunoglobulin isotypes [133] in CD individuals. This reduction may reflect impaired maturation or class switching within the B cell compartment. Another possibility is that circulating memory B cells are recruited to the inflamed gut mucosa, where may participate in immune responses to intestinal bacteria and contribute to the inflammatory environment characteristic of CD. However, an elevated accumulation of memory B cells has not been previously reported in the gut mucosa in CD patients. In gut-associated lymphoid tissue (GALT), only enriched CD27^−^IgD^−^ non-naïve, non-classical switched memory B cells has been observed [133]. Diminished memory B cell populations may provide critical insights into the immunopathogenesis of Crohn’s disease and help predict treatment outcomes. In CD, reduced memory B cells have been associated with the loss of mucosal IgA2^+^ memory B cells [134], a shift toward pathogenic IgG4-producing plasmablasts instead of IgA2-producing one [86,135,136], and a decrease in Bregs [101,137]. These alterations may exacerbate chronic inflammation and predict a diminished response to biologic therapy. Furthermore, a low baseline frequency of memory B cells has also been linked to a higher risk of biologic treatment failure and postoperative recurrence. Therefore, monitoring both the quantity and phenotype of memory B cells could help stratify patients for biologic therapy and predict relapse.

In parallel with the reduction in memory B cells, an increased proportion of CD24^int^CD38^int^ mature naïve B cells has also been reported in the peripheral blood of adult patients with CD [132] as well as in children with CD-both untreated [130] and following conventionally therapy [138]. IL-1β^+^IFNγ^+^TNF-α^+^ naive B cells [139] were likewise found to be increased in the intestinal mucosa of adult CD patients. It is hypothesised that CD may be associated with decreased conversion of naive B cells into memory B cells, or increased production of naive B cells in the bone marrow.

Patients with CD also showed a significant reduction in CD24^hi^CD38^hi^ transitional B cells, which negatively correlated with faecal calprotectin and platelet count—two established markers of mucosal inflammation and disease activity. Transitional B cells exhibit regulatory capacity by producing IL-10 after in vitro stimulation with mitogens [106,107,140]. A reduced pool of circulating transitional B cells has also been reported in paediatric CD patients prior to IFX treatment [138]. However, other studies have observed either unchanged [137] or increased [121] proportions of these cells in newly diagnosed adult CD patients. We suggest that the reduced frequency of transitional B cells may be a feature of the CD patients who are resistant to previous conventional therapy. This decline may be due to their migration to the inflamed intestinal mucosa, although histological evidence supporting mucosal accumulation of transitional B cells in CD remains limited.

We further report a decreased frequency of plasmablasts at baseline. Plasmablasts, the precursors of antibody-secreting plasma cells, migrate to sites of inflammation via the CXCR3 chemokine receptor [139]. Short-lived plasmablasts have also been identified as an important source of IL-10 in inflamed tissue in human disease, including CD [141]. A novel population of FoxP3-expressing CXCR3^+^ plasmablast has been detected in inflamed mucosa of patients with IBD [139]. However, none of the previous studies have examinated plasmablasts in the peripheral blood of CD patients. We speculate that the decrease in circulating plasmablasts may reflect their recruitment and accumulation in the inflamed intestinal mucosa of patients with active CD, where increased numbers of IgA- and granzyme B-producing CD27^+^CD38^high^ B cells have been previously observed [142]. There, they may contribute to modulating the inflammatory response.

A decrease in the frequency of CD5^+^CD1d^+^ B cells in the blood of CD patients prior to the start of biologics was also observed. Additionally, the proportion of these cells negatively correlated with faecal calprotectin. CD5^+^CD1d^+^ B cells represent a unique subset of potent Bregs that controls the T cell-dependent inflammatory response [143]. There have been no studies assessing the pool of CD5^+^CD1d^+^ B cell subset in CD patients. However, CD1d^+^ B cells with low production of IL-10 and TGF-β in the mesenteric lymph of SAMP1/Yit mice with ileitis have been previously reported [144]. We suppose that the decline of CD5^+^CD1d^+^ B cells in CD may be associated with the migration of circulating CD5^+^CD1d^+^ B cells to the inflamed intestine mucosa, where their regulatory function is limited.

Increased surface expression of the CD161 receptor on circulating Tregs was also observed in CD patients prior to the administration of biologic therapy. However, no correlation was found between the CD161 expression on Tregs and standard disease activity markers. Interestingly, a negative correlation was observed between the total frequency of Tregs and CDAI. CD161 is a C-type lectin-like receptor that interacts with its cognate ligand, lectin-like transcript 1 (LLT1), in areas of inflammation. It functions as a co-stimulatory molecule on CD161^+^ Treg cells, and its co-ligation enhances the production of cytokines such as IL-10, IL-17A, IL-22, and IL-4. CD161 expression is induced by all-trans retinoic acid (ATRA) and dendritic cells. While there is a lack of studies assessing the frequency of CD161^+^ Tregs in the peripheral blood of CD patients, the enrichment of this subset has been observed in colonic mucosa. These cells are highly suppressive and have been shown to accelerate wound healing of colonic epithelium [145]. Thus, increased CD161 expression on Tregs in CD patients may enhance both their immunosuppressive and tissue-reparative functions. Although the proportion of CD5^+^ B cells was unchanged in CD patients, a negative correlation with inflammatory markers such as faecal calprotectin and platelet count has also been reported. These findings suggest that circulating CD5^+^ B cell levels may not distinguish CD patients from healthy individuals but could still be relevant to intra-individual variation in disease activity.

Altered B cell subset composition at baseline—characterised by reduced transitional B cells, CD5^+^CD1d^+^, and CD5^+^ B cells, and their negative correlation with faecal calprotectin levels and/or platelet counts—contributes to immune dysregulation and the pathogenesis of CD.

### 3.2. Therapy-Induced Changes in B-Cell Populations and CD161 Expression on Tregs

After induction therapy, patients treated with IFX or ADA exhibited a reduced pool of mature naïve B cells; however, no correlation was observed between mature naïve B cell frequency and biomarkers of systemic and mucosal inflammation. To our knowledge, this is the first study reporting a depletion of naïve B cells in CD patients following anti-TNF-α induction therapy. This shift may reflect improved B cell maturation dynamics in treatment responders.

An increased frequency of CD24^hi^CD27^+^ B cells was also observed in CD patients treated with IFX/ADA. Nevertheless, these cells did not correlate with any disease activity biomarkers. CD24^hi^CD27^+^ B cells represent a major subset of IL-10-producing regulatory B cells. In addition to IL-10 production, they express TGF-β, granzyme B and CD39-an ecto-ATPase [146], and have been shown to suppress CD4^+^ T cell proliferation as well as INF-γ and IL-17 expression. However, studies assessing this B cell subset post-IFX/ADA treatment in CD patients remain limited. Previous investigations reported an enhanced proportion of circulating IL-10-producing B cells at week 14 in adults [101], and increased IL-10-producing CD24^hi^CD38^hi^ transitional B cells after 12 months of IFX treatment in paediatric patients [138]. The changes in the frequency of circulating mature naive B cells and CD24^hi^CD27^+^ B cells at week 14 for IFX and at week 12 for ADA may have potential as a predictive biomarker for treatment response or disease progression in CD, although further longitudinal studies are needed to confirm this.

For the first time, we demonstrated that ADA-treated CD patients showed increased expression of CD161 receptor on circulating Treg cells at week 12. However, this increase did not correlate with any markers of disease activity. This suggests that ADA treatment may enhance the suppressive function of these cells.

At week 14, VDZ-treated patients displayed a high proportion of CD27^int^CD38^hi^ plasmablasts; however, this was not associated with any inflammatory biomarkers. To our knowledge, there are no previous studies evaluating the proportion of circulating plasmablast in CD patients treated with VDZ. Although an increased proportion of plasmablasts was observed in VDZ-treated patients, their lack of correlation with inflammatory markers currently limits conclusions regarding their predictive value. Further longitudinal studies are necessary to determine whether this population could serve as a biomarker for treatment response or disease progression.

### 3.3. Study Limitations

This study has several limitations. Firstly, our CD cohorts following induction therapy were relatively small—consisting of 17 patients in the IFX and ADA groups, and only 7 patients in the VDZ group. Secondly, a notable limitation is the lack of evaluation of the IL-10 producing Bregs, which was not feasible due to the use of TransFix/EDTA blood collection tubes. TransFix is a blood stabiliser that prevents cellular stimulation and, consequently, IL-10 production. Thirdly, all immunophenotyping was performed on peripheral blood samples; therefore, the relevance of our findings to mucosal immune responses remains uncertain, especially considering that many of these populations may preferentially home to inflamed gut tissue. Nonetheless, these limitations do not diminish the overall significance of our findings.

## 4. Materials and Methods

### 4.1. Patients

We recruited adult patients with CD (*n* = 43) from the population based IBD cohort of Gastroenterology Clinic, Jan Biziel University Hospital No. 2, Bydgoszcz, Poland who were qualified for biological therapies as part of the national drug program funded by the National Health Fund.

The diagnosis for CD was made by gastroenterologists based on disease history, exclusion of gastrointestinal infections, endoscopic examination, and pathological assessment of biopsies.

Recruited patients (>18 years) were bio-naïve with mild to severe active CD (Crohn’s Disease Activity Index (CDAI) score > 330). Patients had either lost response or were intolerant to either conventional therapy (steroids and immunosuppressants). Patients fulfilled the criteria and were qualified for biological therapies as part of the program funded by the National Health Fund.

Patients were divided into three groups depending on receiving the biological therapies: first group receiving IFX (*n* = 18) second ADA (*n* = 18) and third one receiving VDZ (*n* = 7). Healthy controls were used as a control group. IFX, ADA and VDZ-treated CD patients as well as healthy controls were matched for gender and age. The characteristic of recruited CD patients and control group were reported in Table 3.

The treatment regimen was administered according to the approved drug. ADA was started by an initial 160 mg subcutaneous (sc) injection followed by 80 mg, and then 40 mg injection every 2 weeks. Regarding IFX, induction therapy was based on intravenous infusions (iv) of 5 mg/kg at weeks 0, 2, and 6, followed by infusions of 5 mg/kg every 8 weeks as maintenance therapy. VDZ was started with 300 mg intravenous infusions at weeks 0, 2, and 6 as induction therapy, followed by 300 mg infusions every 8 weeks as maintenance therapy. If patients respond to induction treatment and obtain a clinical response, they are eligible for maintenance treatment. Treatment induction assessment takes place in week 12 for ADA and in week 14 for IFX and VDZ. Clinical response is defined as a reduction in the CDAI score of at least 70 points and at least 25% from baseline. The drug administration schedules are determined in accordance with the national drug program and the characteristics of the medicinal product.

Prior to the administration of biological medicine, patients underwent a series of physiological assessments, including body temperature, blood pressure, and heart rate monitoring. Patients did not show any symptoms of infection.

Peripheral blood from CD patients was collected twice, before the first dose of biological treatment (at week 0) and after induction therapy (for ADA at 12 week of therapy, for IFX and VDZ at 14 week of therapy). Healthy controls peripheral blood was used as a control.

Exclusion criteria for patients in the CD cohort were cancer diseases, severe kidney or liver diseases. Patients were treated with 5-aminosalicylic acid (5-ASA), corticosteroids and immunosuppressants (mercaptopurine or azathioprine).

All CD patients and controls gave written informed consent to participate in this study. For peripheral blood collection, permission was obtained from the Bioethics Commitee at the Nicolaus Copernicus University (No. 286/2021).

### 4.2. Sample Collection

Peripheral venous blood was collected from each patient into a TransFix/EDTA vacuum tube (Cytomark, Buckingham, UK) before the first infusion of the biologics and after the induction treatment at week 12 and at week 14, depending on the received therapy. The active components of TransFix stabilise leukocytes and their antigens for up to 14 days. Blood was stored at 4 °C until cytometric assays were performed.

### 4.3. Flow Cytometry

**Figure 8:**
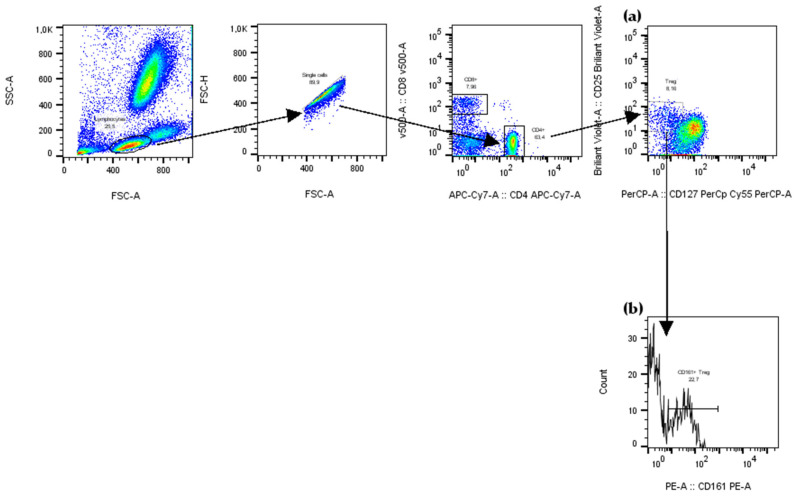
Representative dot plots, showing the gating strategy for flow cytometry analysis of total T regulatory cells (Tregs) and their CD161^+^ subset in the peripheral blood. (**a**) CD4^+^CD25^+^CD127^−^ T cells (Tregs) and (**b**) CD4^+^CD25^+^CD127^−^CD161^+^ T cells (CD161^+^ Tregs) were gated.

**Figure 9:**
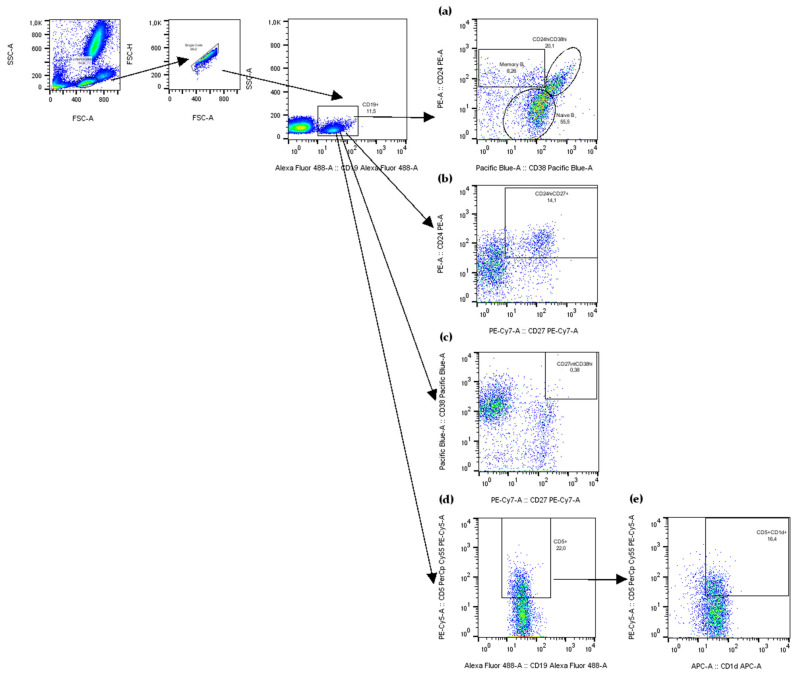
Representative dot plots, showing the gating strategy for flow cytometry analysis of peripheral B cells and their subsets. (**a**) According to CD24 and CD38 expression levels, B cells were sub-gated into: CD19^+^CD24^int^CD38^int^ (mature naïve B cells), CD19^+^CD24^hi^CD38^−^ (memory B cells), and CD19^+^CD24^hi^CD38^hi^ (immature transitional B cells). (**b**) CD19^+^CD24^hi^CD27^+^ B cells (B10 cells) were gated. (**c**) CD19^+^CD27^int^CD38^hi^ B cells (plasmablasts) were gated. (**d**) At first CD19^+^CD5^+^ were gated, (**e**) then CD19^+^CD5^+^CD1d^+^ B cells.

Flow cytometry experiments were performed on a FACS Canto II cytometer (Becton Dickinson, San Diego, CA, USA) and analysed using FlowJo software version 10.8.0 (Tree Star, Inc., Ashland, OR, USA). A 100 μL whole blood was surface stained at RT for 30 min with fluorochrome-conjugated antibodies (BD Biosciences, San Diego, CA, USA, and Beckman Coulter Inc., Brea, CA, USA) as listed in Table 4. Fluorescence minus one or isotype-matched antibodies were used as controls. For erythrocytes lysis, the lysing solution was used according to the manufacturer’s instructions (BD Biosciences, San Jose, CA, USA). The cells were washed twice and resuspended in three drops of phosphate-buffered saline (PBS). 10 000 of SSC^low^CD19^+^ cells or 25 000 SSC^low^CD4^+^ cells were acquired. The frequencies of B (CD19^+^CD24^int^CD38^int^ mature naïve, CD19^+^CD24^hi^CD38^−^ memory, CD19^+^CD24^hi^CD38^hi^ immature transitional, CD5^+^ B1, CD5^+^CD1d^+^, CD24^hi^CD27^+^ B10, CD27^int^CD38^hi^ plasmablasts) and T (CD25^hi^CD127^−^ Tregs and CD161^+^ proinflammatory Tregs) cells subsets, in peripheral blood leukocytes was evaluated based on a multistep cell gating (Figure 8 and Figure 9).

### 4.4. Statistical Analysis

Statistical analyses were performed using STATISTICA software (Statistica version 13; StatSoft Europe, Aschheim, Germany). All continuous variables were tested for normal distribution using the Kolmogorov–Smirnov test. Data were expressed as the medians and quartiles for continuous variables that did not meet standard distribution criteria and proportions for categorical variables. Correlation analyses were performed using Spearman’s rank test. Differences in continuous variables between CD patients groups and control group were assessed using the Mann–Whitney U test. Wilcoxon matched-pairs test was used to test for statistically significant differences in variables between CD patients before and after induction therapy. *p* values < 0.05 were considered statistically significant. Figure 1, Figure 2, Figure 3, Figure 4, Figure 5, Figure 6 and Figure 7 were created using GraphPad Prism version 10.5.0 (GraphPad Software, San Diego, CA, USA), whereas Figure 8 and Figure 9 were created using FlowJo software version 10.8.0 (Tree Star, Inc., Ashland, OR, USA).

## 5. Conclusions

Our findings suggest that induction therapy with IFX or ADA in CD patients is associated with an expansion of circulating CD24^hi^CD27^+^ B cells and a reduction in mature naïve B cells, whereas VDZ therapy is linked to elevated plasmablast proportion. These alterations in peripheral regulatory and effector B cell subsets may reflect differential immunological pathways activated by distinct biologics and could serve as potential biomarkers for predicting treatment response in Crohn’s disease. However, further studies are needed to validate their clinical relevance.

## Figures and Tables

**Table 1:** Disease activity markers of CD patients upon entry into the study.

Parametres	CD Patients (*n* = 43)
CDAI, points	330 [316.0; 381.0]
CRP, mg/L	4.80 [1.60; 17.00]
Faecal calprotectin, μg/g	613.0 [112.0; 2715.0]
Platelet, ×10^9^/L	311 [273.0; 384.0]

Values are median [IQR]. CD, Crohn’s disease, CDAI, CD activity index, CRP, C-reactive protein.

**Table 2:** Disease activity markers of CD patients before biological treatment.

	Before ADA (*n* = 18)	Before IFX (*n* = 18)	Before VDZ (*n* = 7)
CDAI, points	345.50 [313; 377]	330 [317; 384]	332 [328; 381]
CRP, mg/L	3.75 [0.80; 9.0]	4.75 [1.20; 29.20]	9.70 [2.70; 28.60]
Faecal calprotectin, μg/g	493.0 [112; 984]	307.5 [59; 2764]	2715 [613; 3720]
Platelet, ×10^9^;/L	334.0 [269; 355]	297.5 [277; 415]	332 [285; 384]

Values are median [IQR]. CD, Crohn’s disease; CDAI, CD activity index; CRP, C-reactive protein; ADA, adalimumab; IFX, infliximab; VDZ, vedolizumab.

**Table 3:** Characteristics of recruited CD patients and healthy controls.

	CD IFX (*n* = 18)	CD ADA (*n* = 18)	CD VDZ (*n* = 7)	Controls (*n* = 16)
Women	8 (44.8)	10 (55.6)	3 (42.9)	5 (31.2)
Males	10 (55.6)	8 (44.4)	4 (57.1)	11 (68.8)
Age ± SD	37.8 ± 11.9	34.8 ± 9.9	42.6 ± 15.9	40 ± 10.1
Without therapy	1	2	0	-
Therapy with 5-ASA	2	1	1	-
Therapy with budesonide	0	1	0	-
Therapy with prednisone	0	1	0	-
Therapy with immunosuppressants	2	1	0	-
Therapy with 5-ASA + prednisone	3	4	2	-
Therapy with 5-ASA + immunosuppressants	2	1	1	-
Therapy with budesonide + immunosuppressants	0	1	0	-
Therapy with prednisone + immunosuppressants	2	4	2	-
Therapy with 5-ASA + prednisone + immunosuppressants	6	2	1	-

Values are *n* (%) or mean ± SD. CD, Crohn’s disease; IFX, infliximab; ADA, adalimumab, VDZ; vedolizumab; 5-ASA, 5-aminosalicylic acid.

**Table 4:** Fluorochrome-conjugated monoclonal antibodies used in cytometry studies.

	Antibodies
B cell subsets	anti-CD19 FITC (clone 4G7), anti-CD24 PE (clone ML5-RUO), anti-CD27 PE-Cy7 (clone M-T271), anti-CD38 BV421 (clone HIT2), anti-CD5 PerCP-Cyanine 5.5 (clone L17F12), and anti-CD1d APC (clone CD1d42) (all from BD Biosciences)
Regulatory T cells and their subsets	anti-CD4 APC-Cy7 (RPA-T4 clone), anti-CD8 V500 (clone RPA-T8), anti-CD25 BV421 (M-A251 clone), anti-CD127 PerCP-Cyanine 5.5 (clone HIL-7R-M21), anti-CD127 FITC (clone R34.34), anti-CD45RA APC (clone HI100), (all from BD Biosciences). CD161 PE (clone 191B8) (Beckman Coulter)

## Data Availability

The data are available from the corresponding author upon reasonable request.

## Abbreviations

The following abbreviations are used in this manuscript:

ADA	Adalimumab
Bregs	Regulatory B cells
CD	Crohn’s disease
CDAI	Crohn’s disease activity index
CRP	C-reactive protein
IFX	Infliximab
Tregs	Regulatory T cells
VDZ	Vedolizumab
